# Endoscopic Aspects of Gastric Syphilis

**DOI:** 10.1155/2012/646525

**Published:** 2012-08-09

**Authors:** Mariana Souza Varella Frazão, Thiago Guimarães Vilaça, Fred Olavo Aragão Andrade Carneiro, Kengo Toma, Carolina Eliane Reina-Forster, Elisa Ryoka Baba, Spencer Cheng, Thiago Ferreira de Souza, Eduardo Guimarães Hourneaux de Moura, Paulo Sakai

**Affiliations:** Gastrointestinal Endoscopy Unit of the Hospital of Clinics of the University of São Paulo Medical School, Avenue Dr. Enéas de Carvalho Aguiar, 255 6° Andar do Prédio dos Ambulatórios, Cerqueira César, CEP 05403-000, São Paulo, SP, Brazil

## Abstract

*Introduction*. Considered as a rare event, gastric syphilis (GS) is reported as an organic form of involvement. Low incidence of GS emphasizes the importance of histopathological analysis. *Objective*. We aim to characterize GS endoscopic aspects in an immunocompetent patient. *Case Report*. A 23-year-old man presented with epigastric pain associated with nausea, anorexia, generalized malaise and 11 kg weight loss that started 1 month prior to his clinical consultation. Physical examination was normal except for mild abdominal tenderness in epigastrium. Endoscopy observed diminished gastric expandability and diffuse mucosal lesions, from cardia to pylorus. Gastric mucosa was thickened, friable, with nodular aspect, and associated with ulcers lesions. Gastric biopsies were performed, and histopathological analysis resulted in dense inflammatory infiltration rich in plasmocytes. Syphilis serologies were positive for VDRL and *Treponema pallidum* reagents. Immunohistochemical tests were positive for *Treponema pallidum* and CD138. The patient was treated with penicillin, leading to resolution of his clinical complaints and endoscopic findings. *Conclusion*. Diagnosis suspicion of GS is important in view of its nonspecific presentation. Patients with gastric symptoms that mimic neoplastic disease should be investigated thoroughly based on the fact that clinical, endoscopic, and histological findings can easily be mistaken for lymphoma or plastic linitis.

## 1. Introduction

The first reports of syphilis occurred in the 15th century as a sexually transmitted disease. In the early 20th century, it was considered a relevant etiology in neurological and cardiovascular disorders with an increased incidence [1]. 

 From the decade of 50, the advent of penicillin as well as public health measures provided a reduction in disease carriers. A new increase in reported cases was observed in the 80s, after the arise of acquired immunodeficiency syndrome [1].

Syphilis can be divided into three clinical stages that guide its treatment [1].

Although considered a rare event, gastric syphilis (GS) is reported as a form of organic involvement. Andral et al., in 1834, were pioneers by reporting two suspected cases of GS [2]. Other studies, in the next century, described a high incidence of GS diagnosis based on clinical, serological, and radiological evidences [3, 4].

GS first case with histopathological confirmation in surgical specimen was reported by Graham [5]. Later, autopsy studies observed a lower incidence of GS, emphasizing the importance of histopathological analysis [6, 7].

## 2. Objective

We aim to characterize GS endoscopic aspects in an immunocompetent patient.

## 3. Case Report

A 23-year-old non-Caucasian man presented with epigastric pain associated with nausea, postprandial vomiting, anorexia, generalized malaise, and 11 kg weight loss that started 1 month prior to his clinical consultation at Internal Medicine Department of Clinics Hospital of Sao Paulo University.

He had no other important symptoms, and his personal and family antecedents for gastrointestinal disorders, abdominal surgery, other diseases, or previous hospitalization were negative. Physical examination was normal except for mild abdominal tenderness in epigastrium.

**Figure 1:**
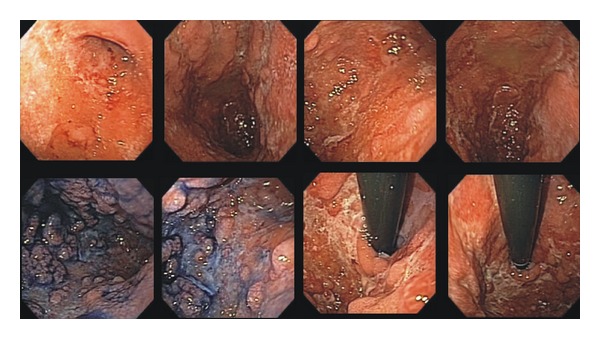
Gastric endoscopic aspects before treatment.

**Figure 2:**
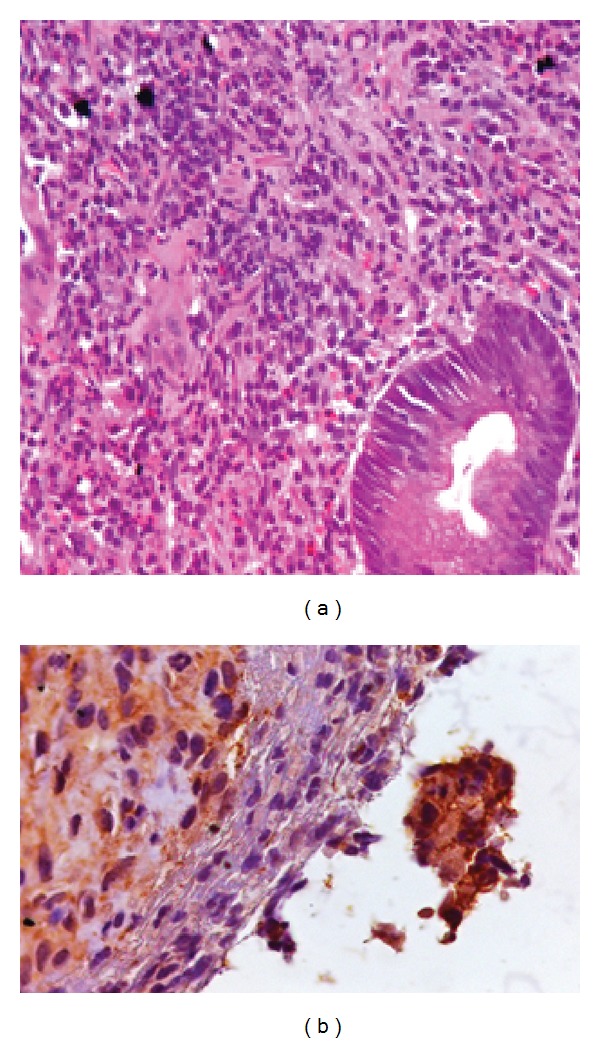
Histopathological study with dense inflammatory infiltration rich in plasmocytes and immunohistochemical test positive for *Treponema pallidum*.

Upper endoscopy observed diminished gastric expandability and diffuse mucosal lesions, from cardia to pylorus. Gastric mucosa was thickened, friable, with nodular aspect, and associated with ulcers lesions (Figure 1). Gastric biopsies were performed, and histopathological analysis resulted in dense inflammatory infiltration rich in plasmocytes (Figure 2).

Laboratory investigations revealed normal hemoglobin, hematocrit, white blood cell count, and liver and kidney function. Serology for HIV was negative. Syphilis serologies were positive for VDRL and *Treponema pallidum* reagents.

Based on laboratory and endoscopic findings, the possibility of GS was raised, and further investigation proceeded with immunohistochemical tests, which were positive for *Treponema pallidum* and CD138 (Figure 2).

**Figure 3:**
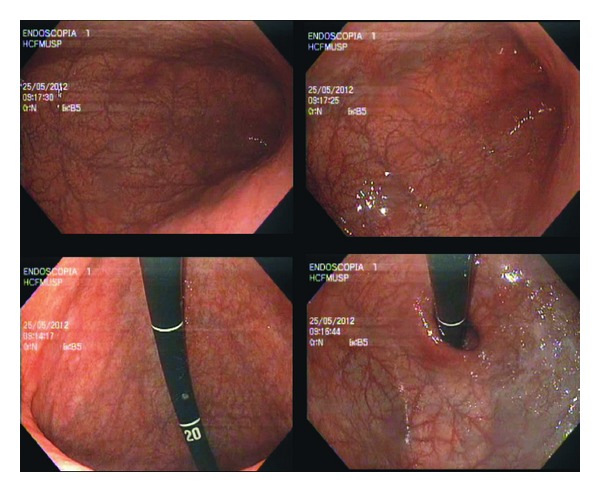
Gastric endoscopic aspects two months after treatment.

The patient was treated with 2.400.000 UI dose of penicillin, leading to resolution of his clinical complaints and endoscopic findings (Figure 3).

## 4. Discussion

The most common symptoms of GS are epigastric pain, anorexia, early satiety, nausea, vomiting, and weight loss [8, 9]. Physical examination frequently does not contribute to the diagnosis [10].

Upper gastrointestinal bleeding often occurs in early stage of disease since that later stage is characterized by being devoid of blood supply to mucosa due to an endarteritis obliterans process. Gastric perforation and obstruction are rare but of serious complications [11–13].

A systematic review published in 2010 showed that the majority of patients with GS had no clinical history (87%) or physical examination (56%) compatible with syphilis. Based on that, proper association between medical and sexual history, physical examination, and especially the high degree of suspicion is required for diagnosis, in view of its difficulty and imprecision [10].

Syphilis serologies are often positive and correlated with the stage of infection. Serological tests include nonspecific and specific [14]. Nonspecific tests are VDRL (venereal disease research laboratory) and RPR (rapid plasma reagin). Specifics tests are FTA-Abs (fluorescent treponemal antibody absorption), TPHA (treponema pallidum haemagglutination test), and ELISA (enzyme-linked immunosorbent assay), in which they use *T. pallidum* antigens [15].

Upper endoscopy usually reveals a diminished gastric expandability. Other findings include mucosal edema, enanthema, friability, erosions, superficial ulcers, nodularity, and hypertrophy of gastric folds [16–22]. Differential diagnoses include lymphoma, plastic linitis, tuberculosis,tic and Crohn's disease [23–27].

Histopathological analysis are compatible with endovasculitis, which includes arterial wall and submucosal layer thickening, perivascular cell infiltrate, diffuse lymphocytic, and plasmocytes infiltrate [8]. Vasculitis, manifested by endarteritis or endophlebitis, is a typical finding in other sites but is rarely observed in gastric samples, probably because endoscopic biopsies do not reach submucosal layer [28].

A finding of chronic inflammatory process similar to what is described the described suggests that syphilis should be investigated as a potential cause [8]. Hematoxylin-eosin analysis may indicate, but does not confirm, diagnosis because *Treponema pallidum* is not identified in this method. In these cases, more specific tests such as immunofluorescence are needed [28].

In summary, diagnosis suspicion of GS is extremely important in view of its nonspecific presentation. Young patients with gastric symptoms that mimic neoplastic disease should be investigated thoroughly based on the fact that clinical, endoscopic, and histological findings can easily be mistaken for lymphoma or plastic linitis.

## References

[1] Hook EW, Marra CM (1992). Acquired syphilis in adults. *The New England Journal of Medicine*.

[2] Morton CB (1932). Syphilis of the stomach. *Archives of Surgery*.

[3] Sexton RL, Dunkley RE, Kreglow AF (1937). Gastroscopic study of 100 cases of early syphilis. *Transactions of the American Therapeutic Society*.

[4] Hartwell JA (1925). Syphilis of the stomach: a critical review of reported cases from the pathological and clinical viewpoints. *Annals of Surgery*.

[5] Graham EA (1922). Surgical treatment of syphilis of the stomach. *Annals of Surgery*.

[6] Symmers D (1916). Anatomic lesions in late acquired syphilis: a study of 314 cases based on the analysis of 4880 necropsies at Bellevue Hospital. *The Journal of the American Medical Association*.

[7] Singer HA, Meyer KA (1929). Syphilis of the stomach with special reference to its incidence. *Surgery, Gynecology & Obstetrics*.

[8] Fujisaki T, Tatewaki M, Fujisaki J (2008). A case of gastric syphilis. *Clinical Gastroenterology and Hepatology*.

[9] Greenstein DB, Wilcox CM, Schwartz DA (1994). Gastric syphilis: report of seven cases and review of the literature. *Journal of Clinical Gastroenterology*.

[10] Mylona EE, Baraboutis IG, Papastamopoulos V (2010). Gastric syphilis: a systematic review of published cases of the last 50 years. *Sexually Transmitted Diseases*.

[11] Winters HA, Notar-Francesco V, Bromberg K (1992). Gastric syphilis: five recent cases and a review of the literature. *Annals of Internal Medicine*.

[12] Vaughan WP, Straus FH, Paloyan D (1977). Squamous carcinoma of the stomach after luetic linitis plastica. *Gastroenterology*.

[13] Morin ME, Tan A (1980). Diffuse enlargement of gastric folds as a manifestation of secondary syphilis. *The American Journal of Gastroenterology*.

[14] Gwanzura L, Latif A, Bassett M, Machekano R, Katzenstein DA, Mason PR (1999). Syphilis serology and HIV infection in Harare, Zimbabwe. *Sexually Transmitted Infections*.

[15] Lautenschlager S (2006). Cutaneous manifestations of syphilis: recognition and management. *American Journal of Clinical Dermatology*.

[16] Abdu RA, Carter K, Pomidor WJ (1993). Gastric syphilis mimicking linitis plastica. *Archives of Surgery*.

[17] Long BW, Johnston JH, Wetzel W, Flowers RH, Haick A (1995). Gastric syphilis: endoscopic and histological features mimicking lymphoma. *The American Journal of Gastroenterology*.

[18] Anai H, Okada Y, Okubo K, Okamura T (1990). Gastric syphilis simulating linitis plastica type of gastric cancer. *Gastrointestinal Endoscopy*.

[19] Prolla JC, Kobayashi S, Yoshii Y, Yamaoka Y, Kasugai T (1970). Diagnostic cytology of the stomach in gastric syphilis: report of two cases. *Acta Cytologica*.

[20] Reid AC, Behan PO (1981). Subacute Wernicke’s encephalopathy due to gastric syphilis. *British Journal of Venereal Diseases*.

[21] Smith MB, Levin TN (1992). Gastric syphilis: an unusual endoscopic appearance. *Gastrointestinal Endoscopy*.

[22] Manten HD, Harary AM, Bockus HL (1985). Chronic infections of the stomach. *Gastroenterology*.

[23] Besses C, Sans-Sabrafen J, Badia X, Rodriguez-Mendez F, Salord JC, Armengol JR (1987). Ulceroinfiltrative syphilitic gastropathy: silver stain diagnosis from biopsy specimen. *The American Journal of Gastroenterology*.

[24] Beckman JW, Schuman BM (1986). Antral gastritis and ulceration in a patient with secondary syphilis. *Gastrointestinal Endoscopy*.

[25] Moore AB, Aurelius JR (1928). Roentgenologic manifestation in eighty-seven cases of gastric syphilis. *American Journal of Roentgenology*.

[26] Jones BV, Lichtenstein JE (1993). Gastric syphilis: radiologic findings. *American Journal of Roentgenology*.

[27] Cecilia M (1999). *Gastrointestinal Pathology, An Atlas and Text*.

[28] Chen CY, Chi KH, George RW (2006). Diagnosis of gastric syphilis by direct immunofluorescence staining and real-time PCR testing. *Journal of Clinical Microbiology*.

